# Functional and environmental performance of plant-produced crumb rubber asphalt mixtures using the dry process

**DOI:** 10.1617/s11527-021-01790-y

**Published:** 2021-10-05

**Authors:** M. Bueno, R. Haag, N. Heeb, P. Mikhailenko, L. Boesiger, L. D. Poulikakos

**Affiliations:** 1 Empa, Swiss Federal Laboratories for Material Science and Technology, Überlandstrasse 129, 8600 Dübendorf, Switzerland; 2 Ammann Schweiz AG, Langenthal, Switzerland

**Keywords:** Crumb rubber, Asphalt mixture, PAH, VOC emissions, Traffic simulator, Surface texture, MMLS3

## Abstract

Incorporating crumb rubber (CR) using the dry process, directly in the asphalt mixture rather than into the bituminous binder requires no plant retrofitting, and therefore is the most practical industrial method for CR incorporation into asphalt mixtures. Nevertheless, very few large scale studies have been conducted. This work uses a holistic approach and reports on the functional and environmental performance of asphalt mixtures with different concentrations of CR fabricated employing the dry process in asphalt plants. Gaseous emissions were monitored during the production and laboratory leaching tests simulating the release of pollutants during rain, was conducted to evaluate the toxicology of both the CR material alone and the modified asphalt mixtures. In addition, laboratory compacted samples were tested to assess their fatigue behavior. Furthermore, noise relevant surface properties of large roller compacted slabs were evaluated before and after being subjected to a load simulator (MMLS3) to evaluate their resistance to permanent deformation. The results confirm that comparable performance can be achieved with the incorporation of CR using the dry process for high performance surfaces such as semi-dense asphalt, which usually require the use of polymer modified binders. Environmental performance improvement can be achieved by a washing step of the CR material that could remove polar CR additives which have commonly been used as vulcanization accelerator during rubber production.

## Introduction

Crumb rubber (CR) from waste tires has been used as modifier of asphalt materials for the last 40 years [1]. For the blending process various methods have been developed over the years that can be summarized under three general categories. First, is the wet process that refers to the blending of CR with bitumen and added to the asphalt mixture after a reaction time; second, is the dry process where the CR is added directly to the mixture and third the terminal blend that refers to bitumen with CR which is digested into the bitumen at the refinery or at a bitumen terminal. Many practical studies have shown that the use of CR can improve the properties of asphalt mixtures as a result of interaction withthe bituminous binder. The main physical mechanism governing this interaction involves a swelling process of the rubber particles by the lower molecular weight fractions of the binder (e.g. maltenes) [2]. Nevertheless, the final performance of this solution is closely linked to a good mixture design including percentage of binder and CR. Along with the selection of a compatible asphalt binder, the type of CR particle consequence of its production plays a key role in obtaining a reliable behavior of the final asphalt mixture [3].

Nowadays, the dry manufacturing process seems to be the technique that is more practical to use for the industry, as it does not require significant changes in the production plants. Beside the practical knowledge gained, the development of chemical treatments focused on the enhancement of the compatibility between the CR and the asphalt binder has led to overcome the initial inconsistencies found during the first test trials [4]. These surface treatments can involve catalytic agents, dispersing agents, and hydro-thermal reactions to activate CR particles [2]. As a consequence of this, CR particles can now be added directly to the mixer as additive or partial replacement of the mineral aggregates. After mixing with the optimal amount of hot bitumen (ca. 160–220 °C), the mixture usually requires a curing, maturation or digestion time at high temperature to achieve a proper reaction between the CR particles and the asphalt binder. The nature of this interaction is a combination of physical and chemical as reported in a review by Lo Presti [5] comprising partial digestion of the rubber into the bitumen on the one hand and, on the other, adsorption of the aromatic oils within the polymeric chains that are the main components of the rubber. Latest versions of modified CR have reduced this time to 30 min or lower, which could easily coincide with the hauling distance from the plants to the construction site [6].

Most of the published works about the performance of CR asphalt mixtures modified astofiusing the dry process focus on laboratory scale studies [4, 7, 8]. However, very few report on the performance of optimized designs produced at large industrial scale asphalt plants. For example, Feiteira Dias et al. [9] evaluated the mechanical response of gap-graded asphalt rubber mixtures manufactured in an asphalt plant using the dry process in a field study of trial sections. The laboratory testing indicated that the mixtures with CR showed better rutting and fatigue performance. Moreover, visual inspection after five years of service confirmed a satisfactory performance, both with regard to the structural and functional performance. Nevertheless, due to the elevated binder content used in these experimental mixtures (> 8.5%), it was difficult to link this enhanced response only to the addition of the CR. Likewise, Eskandarsefat et al. [10] have shown the effect of CR addition with the dry process on dense asphalt mixtures with reclaimed asphalt pavement (RAP) by means of both laboratory-scale and in situ tests. They aimed at studying the influence of the elastic CR particles on the stiffening effect usually associated to the presence of aged binder from the RAP. Moreover, the potential absorption of the rejuvenating agent by the unmodified CR was analyzed. They observed that CR modified asphalt mixtures showed an optimal response against permanent deformation and moisture susceptibility. However, their results confirmed that the binder content needed to be slightly increased in the design of the CR modified asphalt mixtures to enhance the workability and to meet the volumetric requirements. In addition, the skid resistance measured on the test tracks was found to be reduced with CR which was also found by Miró et al. [40] in the evaluation of the functional characteristics of several test tracks built with gap-graded asphalt mixtures modified with CR by the dry process. Specifically, the addition of CR using the dry process led to a decrease in macrotexture, which was proportional to the increase in CR content. Although it was observed that these mixtures were more prone to wear, they emphasized that construction and service conditions could strongly affect the surface characteristics. In a more recent study, Sangiorgi et al. [11] carried out a field evaluation of the viability of Stone Mastic Asphalt (SMA) mixtures with CR incorporated as partial replacement of limestone filler. They have found that the modified mixtures fabricated in the plant obtained similar volumetric and mechanical properties compared with the standard mixtures. Moreover, although the texture values of these rubberized surfaces were in line with the parameters typically recorded for gap-graded mixtures, they showed lower tire-road noise levels.

In addition to the field performance, the environmental effect of such mixtures is a debate that is going on today in all construction sectors and in fact, has become central for the assessment of new asphalt mixture designs, including the ones modified with CR by the dry process. CR is a mixture of natural and synthetic rubber (such as styrene-butadiene-rubber), carbon black, sulfur and sulfur-based cross-linking agents and various other additives (e.g. aging retardants, reinforcing agents, accelerants, antioxidants, plasticizers, fillers, or textiles) [2]. Due to its constituents, monitoring of the environmental effects of CR is important. In an earlier technical report, [12] showed higher concentrations of diverse pollutants in air samples taken during the production of CR asphalt mixtures as compared with conventional ones. They noted that the presence of polycyclic aromatic hydrocarbons (PAHs) in the modified mixtures could have the potential to cause health problems (e.g. cancer or respiratory irritation) in workers due to occupational exposure. However, in a more recent study, Nilsson et al. [13] concluded that it was not evident if exposure to rubber bitumen possesses a higher risk than exposure to standard bitumen, in terms of air pollutants such as benzothiazole- and PAH-emissions. Likewise, Sangiorgi et al. [11] did report effective benefits for rubberized mixtures with a reduction in air emissions of respirable dust particles and PAHs during the placement process. Also in Italy, Zanetti et al. [14] investigated the gaseous emissions produced during paving operations of asphalt mixtures modified with CR by the dry process. For the determination of the concentration of volatile organic compounds (VOCs) and PAHs, different analytical tests were conducted in the laboratory. They concluded that composition of fumes was affected by several material specific (i.e. mixture composition, CR type and base bitumen type) as well as site-specific (i.e. layer thickness, placement and air temperature, wind, air pressure) factors. Nevertheless, relative contributions of bitumen quantity, type and composition, seem to be the most relevant parameters. Moreover, the results showed that the toxic and carcinogenic risks for workers on site in the case of bituminous mixtures containing CR, were comparable to that of standard paving materials. Along with air pollutant emissions during the construction of different test tracks*, *Santagata et al. [15] quantified the concentration of PAHs, VOC and metals by means of leaching tests. The results confirmed that the values obtained for CR asphalt mixtures complied with the required regulations. The same conclusion had been already reported in several American studies conducted in order to evaluate the potential leachate of hazardous components presented in crumb rubber [16–18]. The authors concluded that the CR modified asphalt mixtures did not show a relevant threat for the environment and human health either.

In an updated review, Wang et al. [19] summarize the overall environmental impact associated to the CR modified asphalt mixtures. The report states that the rubberized asphalt technology was favorable to reducing greenhouse gas GHG emission [20]. Also, the authors note the study by Stout et al. [21] that mention the emissions of O_2_, N_2_, CO_2_, NO_x_ and SO_2_ from the production of rubberized asphalt mixtures were similar to those for hot mix asphalt. However, emissions of CO and CH_4_ were much lower from rubberized asphalt mixtures ca. 40% and 60% respectively. It should be noted that these were measured during the wet process for a continuous manufacturing process. In that review, it is also emphasized that, in their work, Feraldi et al. [22] stated that the use of commercial asphalt modifier based on recycling scrap tires showed excellent environmental advantages and reduced the volatile organic compounds (VOCs) by 30% in comparison with SBS modified asphalt.

With the current state of the art, many researchers consider that asphalt mixtures modified with CR by the dry process can potentially be a substitute for polymer modification. These high performance polymer modified mixtures were originally developed to improve the mechanical behavior to meet the requirements of a growing traffic demand observed during the past decades. However, issues related to their high cost as well as to production temperatures (i.e. greenhouse gas emissions) and recyclability of polymer modified mixtures have been a topic of discussion within the international community.

In spite of the vast amount of knowledge on the use of CR as a performance enhancing additive in road materials and an effective waste mitigation measure, the technology readiness level (TRL) is varied worldwide. For example Piao et al. [23] have shown that wet process CR has reached a TRL of 7–9 (application is partially or completely industrialized) whereas in Switzerland it has reached a TRL of only 1–4 (progress at laboratory scale or lower). On the other hand, the dry process CR has a worldwide TRL level of 5–7 (pilot projects have been implemented in the field) whereas the Swiss TRL level in only 1–4. This is a typical trend that is observed i.e. technologies reaching different levels of TRL. The worldwide discrepancy is due to various factors including lack of legislation and incentives to lack of know-how and trust of the new technologies by the practicing professionals and decision makers. The current work aims at closing this knowledge gap by using a holistic approach by analyzing the functional performance and environmental effects of plant produced asphalt mixtures modified with CR using the dry process in comparison with a conventional one prepared with polymer modified bitumen (PmB). Three batches of semi-dense asphalt mixtures were produced in an asphalt plant where VOC emissions were measured. Afterwards, samples were taken from each mixture in order to conduct a series of leaching tests. Additionally, fatigue tests were conducted on cylindrical samples, while large slabs were roller compacted and used for the evaluation of their surface texture characteristics as well as their responses to permanent deformation at medium scale under repetitive loading with a load simulator.

## Experimental methodology

### Materials

A semi-dense asphalt mixture with a maximum aggregate size of 4 mm (SDA4) was selected for this study. This type of mixture is used commonly as a low noise surface course (*SNR 640 436: 2015*). A total of three 800 kg batches were manufactured in an asphalt plant. The one produced with PmB 45-80-65 was the reference following the Swiss standard. In parallel, a base asphalt binder type 70/100 was used for the mixtures that incorporated different percentages of CR by the dry process directly in the asphalt mixer with the pre-heated mineral fractions. The CR particles (< 0.6 mm) were produced by mechanical shredding and chemically activated by a patented treatment for use in the dry process [24].

While the CR particles could occupy some space in the mixture, no reduction was made in the aggregates or binder to compensate for this. However, a slight increase of the asphalt binder content based on the CR percentage was recommended by the CR producer. This was aimed at countering the potential swelling process that generally involves the absorption of lighter fractions of the asphalt binder into the internal matrix of the rubber particles. The details of the mixture designs are shown in Table 1. The operating parameters, mixing times and temperatures were the same within each mixture manufacturing process. Furthermore, a digestion time of 30 min at the mixing temperature (160 °C) was used for allowing a proper CR-asphalt binder interaction. When working with chemically activated crumb rubber, as here, swelling will be much faster hence the material will reach a state at which it can be paved quicker. This is evidenced in the viscosity measurements over time performed previously [6]. The rubber amount was based on ca. 15% of fresh bitumen as shown by previous experiments [6]. Due to the potential ageing effects on the mixture induced by this stage, and to allow proper comparison between the mixtures, the reference mixtures were subjected to the rest period at high temperature (digestion process) as well. During the production process this CR-asphalt binder interaction was undertaken in silos at the plant site. The mixtures were thereafter placed in 25 kg boxes and transferred to the laboratory for further testing. Prior to the sample preparation, the mixtures were heated briefly in a microwave oven to attain workability.

**Table 1:** Design of the SDA4 mixtures

Description	Bitumen content (wt%)	Binder type	Air void content (vol%)	CR (wt% mixture)
SDA-Ref	6.20	PmB 45–80–65	13.7	–
SDA-0.7% CR	6.52	70/100	14.3	0.7 (10.7 wt% of bitumen)
SDA-1.0% CR	6.63	70/100	13.5	1.0 (15.1 wt% of bitumen)

### VOC emissions

During the plant production process, which is a batch process, the temperatures of the standard materials (mineral aggregates, filler and asphalt binder) were the same for the three different mixtures. This was employed to isolate the effect of the CR that was incorporated at ambient temperature directly into the mixer before the addition of the hot bitumen. No significant difference in terms of handling and mixing was noticed between the mixtures and approximately a temperature of 160 °C was always recorded at the exit of the mixer.

Likewise, in order to assess the effect of the CR incorporation on the concentration of VOCs during the production of the mixtures, samples of emissions were taken at two locations from the mixer aspiration duct and from the exhauster (downstream) in order to quantify the VOCs. The measurements were carried out with a Flame Ionization Detector (SICK FID 3006). This equipment was used for measuring Total Organic Compounds (TOC) or VOCs counted on carbon atoms (VOC-C1).

**Fig. 1:**
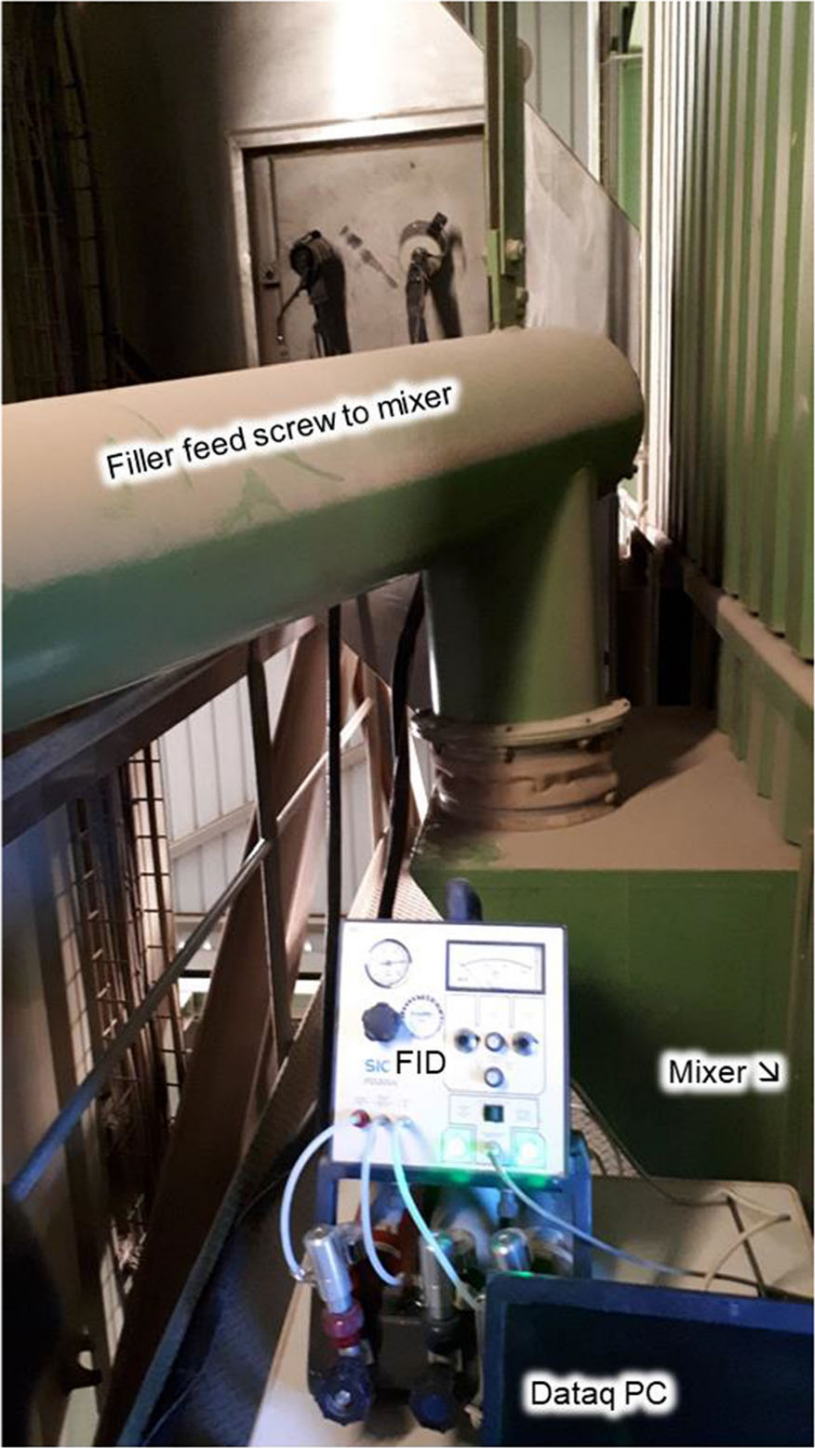
Details of mixer VOC measurement setup at Weibel Oberwangen plant. Sensors were placed **a** in the mixer aspiration duct, and **b** in the clean gas duct downstream of the plant’s exhauster (which is located upstream of the plant’s chimney (or stack))

The sensors were installed at two locations: in the mixer aspiration duct and in the clean gas duct i.e. downstream of the plant’s main bag filter (Fig. 1) and exhauster (main blower) which are located upstream from the exhaust gas chimney. All emission data was recorded with a mobile NI (National Instruments) LabVIEW SignalExpress using a sampling rate of 1 data point per second for each signal (s^−1^).

### Leaching tests

As mentioned earlier, CR is a mixture of natural and synthetic rubber (such as styrene-butadiene-rubber), carbon black, sulfur and sulfur-based cross-linking agents and various other additives (e.g. aging retardants, reinforcing agents, accelerants, antioxidants, plasticizers, fillers, or textiles) [2]. In particular, CR can contain various benzothiazole (BT) derivatives, which are sulfur-based compounds that are used as vulcanization accelerator and/or are by-products of the accelerators formed during the tire vulcanization. Therefore, in asphalt mixtures modified with CR, the release of these compounds in fumes during road work and in run-off water during service life can lead to exposure of humans and the environment to carcinogenic and toxic compounds. Benzothiazole (BT) derivatives are designed to be thermally labile and as such decompose at elevated temperatures e.g. during vulcanization of rubber and road work. These BT decomposition products in the fumes were not specified. However, BT derivatives are relatively stable under ambient temperatures and can be dissolved in water, especially in acidic water and with this be released from CR-modified asphalt. Likewise, any concentration of PAHs has the potential to cause cancer in workers exposed to its fumes during the production of the asphalt mixtures or the final construction of the road infrastructure.

In order to assess concentrations of PAHs and benzothiazoles in the CR material, samples of the CR used in this study were extracted with dichloromethane (DCM) to dissolve the PAHs and BTs. All SDA mixtures produced in the plant, were analyzed in the laboratory by means of a set of leaching tests with acidic water (diluted acetic acid, pH 4.93) using established methods [25]. The materials for leaching tests were prepared in triplicates. For each test, 100 g samples of loose asphalt mixture were placed in a glass bottle and 2 l of acidic solution (pH = 4.93) was added. Mild acidic conditions simulate leaching with rain, which is slightly acidic too. The bottle was rotated at 30 rpm for 18 h at room temperature. Then the extract was separated from the solid using a borosilicate glass fiber filter (0.6–0.8 μm). The PAHs in the leachates were extracted by using solid-phase extraction disks (ENVITM-18 DSK SPE disks, Supelco). A high resolution combined gas chromatograph mass spectrometer (GC-Ultra-HRMS Orbitrap QExactive) was used to identify PAHs by following an established leaching procedure [26]. Concentrations of BT-derivatives were determined with a combined liquid chromatography-triple-quadrupol mass spectrometer (LC-QQQ-MS, Agilent 1290 Infinity). A list of the investigated compounds, abreviations used, molecular masses and if available, water solubilities [27] are given in Table 2.

**Table 2:** Compounds investigated in leachates of crumb rubber-modified asphalts

Compound	Abreviation	Formula	Molecular mass	Water solubility^a^ g/mol mg/L
*PAHs*				
Naphthalene	NAPH	C10H8	128.0621	31.6
Acenaphthylene	ACY	C12H8	152.0623	3.9
Acenaphthene	ACN	C12H10	154.0778	3.5
Fluorene	FLN	C13H10	166.0778	> 0.19
Phenanthrene	PHEN	C14H10	178.0777	1.18
Anthracene	ANTC	C14H10	178.0777	> 0.004
Fluoranthene	FLT	C16H10	202.0777	0.26
Pyrene	PYR	C16H10	202.0777	0.013
Benzo(a)anthracene	BaA	C18H12	228.0934	0.014
Chrysene	CHR	C18H12	228.0934	0.002
Benzo(b)fluoranthene	BbFLT	C20H12	252.0934	0.0012
Benzo(k)fluoranthene	BkFLT	C20H12	252.0934	0.00055
Benzo(a)pyrene	BaP	C20H12	252.0934	> 0.0038
Indeno[1,2,3-cd]pyrene	IP	C22H12	276.0939	0.062
Benzo[ghi]perylene	BghiPER	C22H12	276.0939	0.0003
Dibenz[ah]anthracene	DBahA	C22H14	278.1090	0.0006
*BTs*				
Cyclohexyl-amino-benzthiazole	HABT	C13H16N2S	232.3488	n.d.a
2-(4-Morpholino)-benzthiazole	MoBT	C11H12N2OS	220.2946	n.d.a

n.d.a. no data availible

^a^ From [27]

### Resistance to fatigue

Cylindrical Marshall specimens of 100 mm diameter and 40 mm thickness were prepared by hammer compaction and used to perform indirect tensile tests to obtain the fatigue resistance (EN 12,697-24, AL-SP-Asphalt 09). During the fatigue test until failure, a continuous sinusoidal load was applied with a frequency of 10 Hz at a constant temperature 10 °C. According to the standard, three loading strain amplitudes were implemented using 0.035 MPa as the lower stress and an upper stress between 0.4 and 0.8 MPa.

The material’s fatigue function is expressed as:

1 $$N_{{{\text{Macro}}}} = \,C_{{1}} \, \cdot \varepsilon_{{{\text{el}}}}^{{C_{2} }}$$

where *ε*_el_ is the horizontal elastic initial strain and *C*1, *C*2 are fitting constants. *N*_macro_ is the number of loading cycles when the energy ratio (ER) reaches its peak, N being the number of cycles and *E(N) the* stiffness modulus at each cycle:

2 $${\text{ER}}(N) = \left| {E(N)} \right|N$$

### Permanent deformation with model mobile load simulator MMLS3

**Fig. 2:**
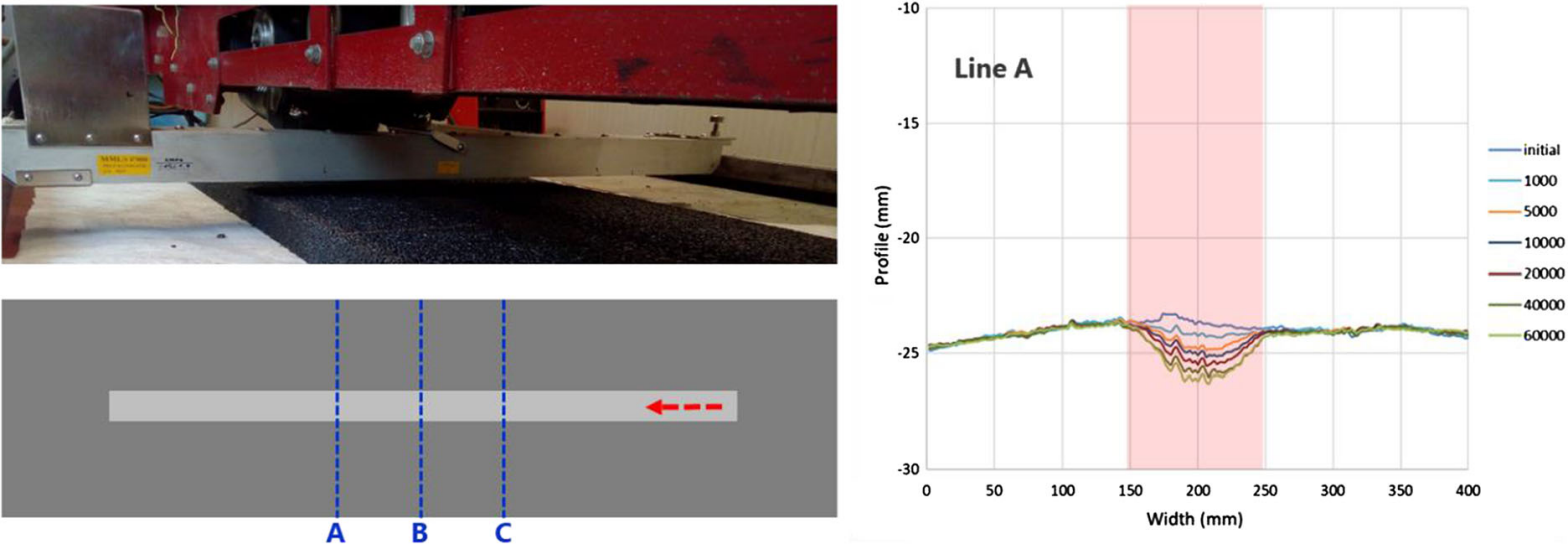
Details of the experimental set up for the MMLS3 tests left top deformation measurement, bottom 3 locations (**a**, **b**, **c**) of the measurements. Right, sample of the profile evolution during different loading cycles

In order to reproduce stresses and strains that are similar to those produced in real pavements, the model mobile load simulator MMLS3 was used as a medium scale accelerated pavement loading testing system [28]*.* Permanent deformation tests at 25 °C were conducted on large slabs (two each) from each asphalt mixture that were roller compacted. These were 1600 mm in length and 435 mm in width with a thickness of 40 mm. During the test, the evolution of the vertical deformation of the rut generated on the wheel path was monitored with a laser scanner with a precision of 1 mm (Fig. 2). Three reference lines transversal to the wheels direction (A, B and C) were scanned at 1000, 5000, 10,000, 20,000, 40,000 and 60,000 loading cycles. The percent rut depth was calculated as an average of the measured points on the rut width (100 mm) at the reference lines resulting in 3 locations and 100 points per location (A, B, C in Fig. 2) resulting in 300 measurements points as follows:

3 $${\text{Rut}} \,{\text{depth }}\left( \% \right) = \frac{{\overline{d}_{n} \left( {{\text{mm}}} \right)}}{{h \left( {{\text{mm}}} \right) }} \cdot 100$$

4 $$\overline{d}_{n} \left( {{\text{mm}}} \right) = \frac{{\mathop \sum \nolimits_{{}}^{{}} d_{x,y,n} \left( {{\text{mm}}} \right) - d_{{x,y,{\text{initial}}}} \left( {{\text{mm}}} \right)}}{3 \cdot 100}$$

where *d*_*x,y*_ are the punctual vertical displacements in *mm* measured along the width of the rut (*x*-direction) on each reference line (*y*-direction) along lines *A*, *B*, *C* (refer Fig. 2) before the test (initial) and after different number of cycles (*n*), $\overline{d}_{n}$ is the average of vertical displacement at each cycle in mm and *h* is the thickness of the slab in mm.

### Surface texture and skid resistance

The texture of the surface is usually associated to the driving safety under wet conditions, noise emission or fuel consumption [29]. In addition, it plays a relevant role in wearing of the rolling tires [30]. This results in tire dust that could potentially have a detrimental effect on the surrounding environment by means of soil and groundwater leachates as well as ultrafine particles suspended in air affecting humans cardiovascular and respiratory systems. Nevertheless, previous research has shown a positive influence of porous pavements in mitigating resuspension of road particles [31]. In this study, the surface properties of the SDA slabs were investigated through their micro- and macro-texture characteristics.

The friction/adhesion between tires and the rolling surface is related to micro-texture. The skid resistance for each slab was measured by using a British pendulum arm tester on wet surfaces at three different pendulum swings on the same location (EN 13,036-4). The tester incorporates a spring-loaded slider made of a standard rubber which upon releasing passes over the test surface. This contact results in an energy loss that is quantified by the upswing of the arm using a calibrated scale. The Pendulum Test Value (PTV) was determined by the constant value achieved by the final three swings.

In addition, the surface macrotexture (texture wavelengths 2.5–100 mm) was measured with stationary laser profilometry (Ames Engineering 9400HD) mounted on top of the asphalt slabs. Texture levels (in dB) were obtained by scanning the profile of the surface. Two different punctual measurements (100 mm × 50 mm) were carried out with resolutions of 0.005 mm vertically, 0.006 mm along the length of the scan and 0.02 mm for the width. Afterwards, the mean profile depth (MPD) was calculated (ISO 13473–2) as the average depth of the surface over a 100 mm baseline. The texture level (*L*_TX,λ_) relative to the texture wavelengths, *λ*, is calculated by taking the 1/3rd octave band power spectral density (PSD) graphs for every 10 scanlines and using the following equation derived from ISO 13473-4:

5 $$L_{{{\text{TX}},\lambda }} = 10{\text{log}}\left( {\frac{{{\text{Z}}_{p,\lambda } *0.232f}}{{a_{{{\text{ref}}}}^{2} }}} \right) {\text{dB}}$$

where ${\text{Z}}_{{p,{ }\lambda }}$ is the 1/3rd octave band PSD amplitude for a certain texture bandwidth, $\lambda$. 0.232f represents the bandwidth, and $a_{{{\text{ref}}}}$ is the reference value of the surface profile amplitude (10^−6^ m given by ISO 13473–4). In addition, an extra set of measurements was conducted on the rolling path after the MMLS3 test in order to evaluate the surface evolution after the wearing caused by the rolling of tires. Two measurements before and after wearing, respectively, were conducted for each sample.

## Results and discussion

**Fig. 3:**
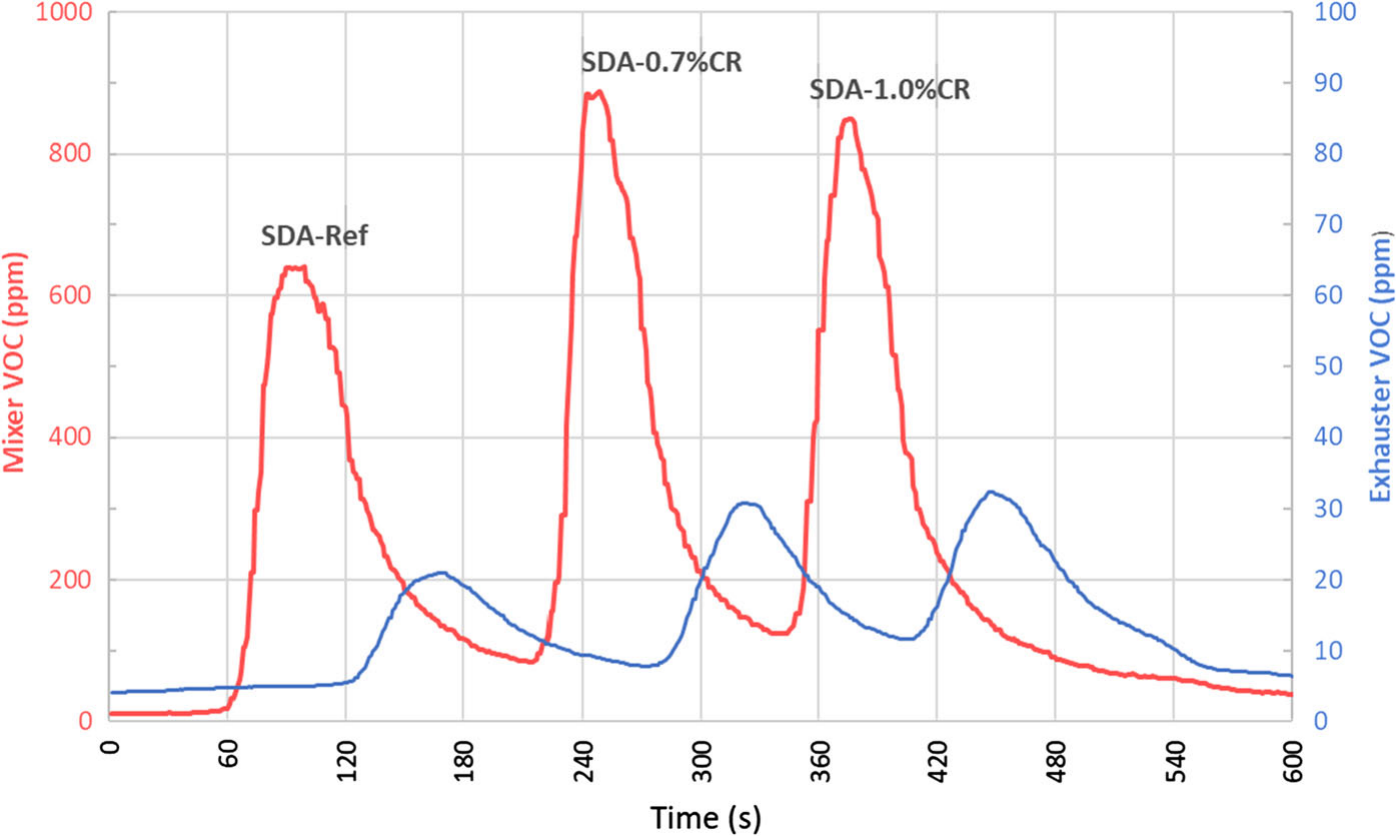
Mixer and exhauster VOC emission levels for the Weibel Oberwangen plant (refer to Fig. 1)

The VOC emissions were measured at the mixer and the chimney during the production of the different SDA mixtures at the Weibel Oberwangen trials. The obtained values are shown in Fig. 3. As expected, the concentration observed at the chimney was lower in comparison to the one measured directly from the mixer. As the graphs show, the concentrations at the exhauster were much lower (see scaling in Fig. 3). In fact, the levels at the blowers were minor due to significant dilution with fresh air sources from the total plant process. In general, VOC peak height concentrations were found to be higher during the production of the experimental CR mixture batches than during the mixing of the reference polymer modified mixture.

To further evaluate these results, the emissions obtained at the mixer are next analyzed in detail. The areas corresponding to each mixture production were calculated. The calculation of the areas allow to consider the entire VOC vs time curve and not only the peak. In this case, a base line correction was applied first to accurately define the accumulated concentration during the slightly different mixing times. Although, a lower peak was observed for the reference mixture that could be related to the slightly longer mixing time used to fabricate the reference mixture (Fig. 3), comparison of VOC areas similar values of 35′243, 35′045 and 32′483 ppm.s were obtained for SDA-ref, SDA-0.7%CR and SDA-1.0%CR respectively.

Furthermore, it is important to note that this evaluation was based on small single batch productions (800 kg) for one type of mixture and one type of plant. In normal operating conditions hundreds of tons are produced daily. Therefore, these results must be only considered as a preliminary evaluation and future measurement campaigns with an increased number of batches should be undertaken for a more solid conclusion and statements. These could further assess other contributing factors such as plant type, schematic and layout, airflows, various mixture designs and mixing sequences, bitumen types and qualities as well as additives (e.g. CR types and addition rates). It should be further noted that mixer emissions are one of many contributors resulting in the total plant exhaust gas emissions measured in the chimney, the latter being relevant for compliance with exhaust gas emission limits. Other emission sources would be burners, transfer points or aspiration systems.

**Fig. 4:**
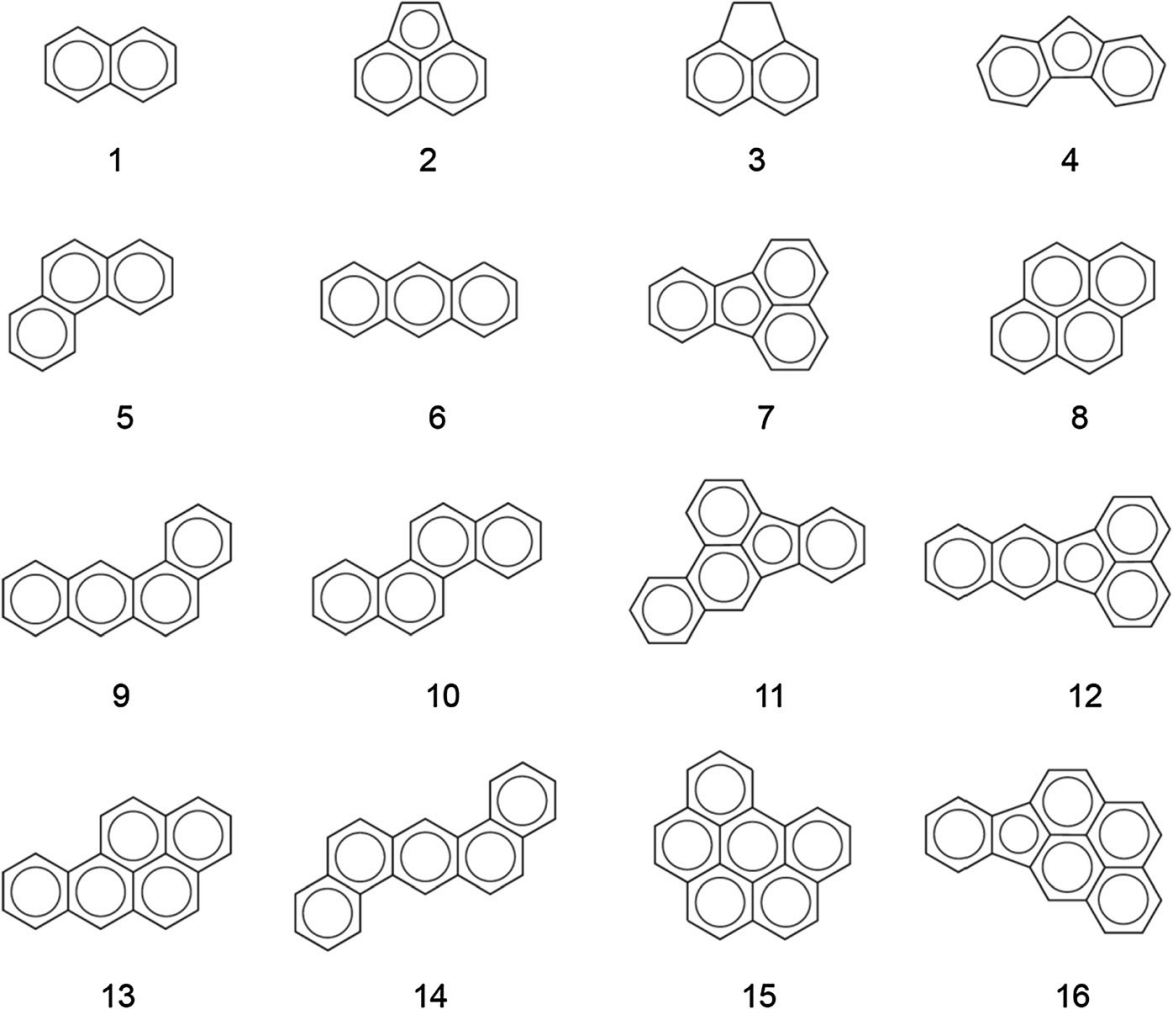
Chemical structures of 16 priority PAHs found in CR materials and leachates from CR-modified asphalts. Naphthalene (1), acenaphthylene (2), acenaphthene (3), fluorene (4), phenanthrene (5), anthracene (6), fluoranthene (7), pyrene (8), benzo(a)anthracene (9), chrysene (10), benzo(b)fluoranthene (11), benzo(k)fluoranthene (12), benzo(a)pyrene (13), dibenz[ah]anthracene (14), benzo[ghi]perylene (15), indeno[1,2,3-cd]pyrene (16) were studied. For abbreviations refer to Table 2

The presence of PAHs in the CR used as modifier and in the different SDA mixtures prepared for this investigation was evaluated and the release of PAHs from these materials to the environment was studied by leaching tests with acidic water. Figure 4 displays chemical structures of 16 priority PAHs. Abbreviations are given in Table 2.

**Fig. 5:**
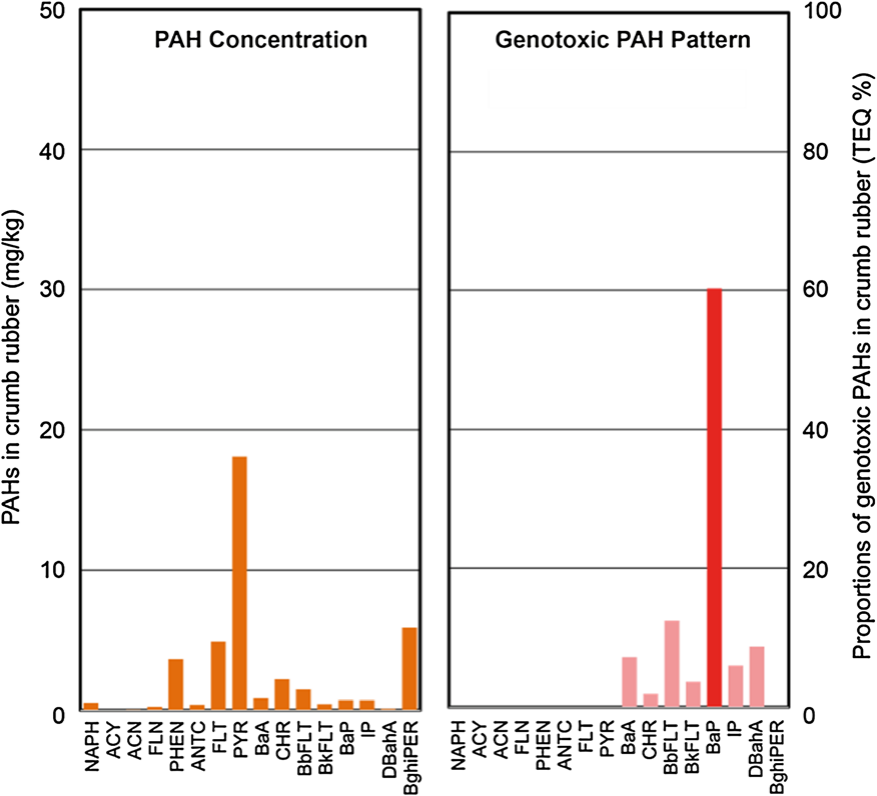
Concentrations (mg/kg) of 16 priority PAHs in DCM-extracts of the CR material (*n* = 1) and pattern of the toxicity-equivalent-weighted genotoxic potential (TEQ-%). For abbreviations refer to Table 2

Concentrations of DCM-extractable PAHs (mg/kg) of the CR material alone are shown in Fig. 5. As reported before [32], pyrene (8) was found to be the main compound within the CR sample followed by benzo(ghi)perylene (15), fluoranthene (7) and phenanthrene (5). The priority PAH concentration of 40 mg/kg CR was significantly lower than the German and Swiss limit values of 250 and 5000 mg/kg for binder materials, respectively [33, 34]. Regarding the genotoxic potential, the toxicity equivalence-weighted (TEQ) sum of carcinogenic PAHs was calculated. With this approach, the importance of the carcinogenic compounds such as benzo(a)pyrene (13), which has the highest genotoxicity factor of 1.0, are better evaluated and the toxicity of CR is found at a concentration of 1.2 mg TEQ/kg. The TEQ-weighted pattern of the genotoxic PAHs is also shown in Fig. 5.

**Fig. 6:**
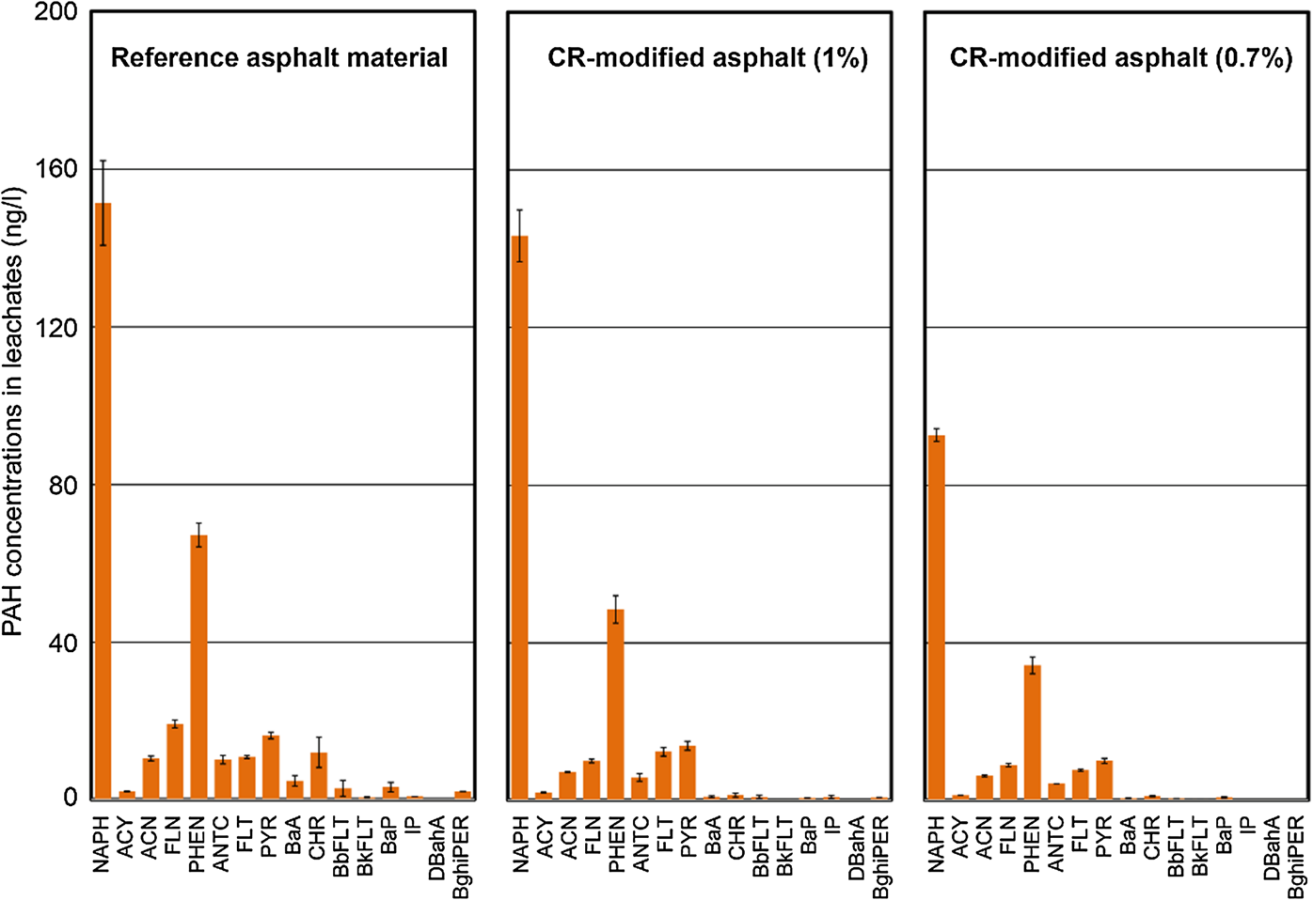
Concentrations (ng/l) of 16 priority PAHs in leachates (*n* = 3). Leachates from reference asphalt without and modified asphalt with 1% and 0.7% CR are compared. For abbreviations refer to Table 2

Leachate concentrations (ng/l) of 16 priority PAHs from different asphalt mixture samples are shown in Fig. 6. It can be observed that leachates from all asphalt mixtures present similar patterns which are directly related to the asphalt-PAH pattern rather than to the CR-pattern (Fig. 5). Comparison of Figs. 5 and 6 shows that the addition of CR had a small effect on the PAH leachate pattern (Fig. 6). Mainly 2- and 3-ring PAHs, which are water soluble to some degree (Table 2), were released from the asphalt mixtures and accumulated in all leachates. The PAH concentrations in the experimental mixtures with CR of 248 ± 16 and 168 ± 6 mg/l were lower in comparison to the concentration obtained for the reference mixture prepared with PmB (314 ± 26 mg/l). The extraction experiments were repeated three times (n = 3) and produced reproducible results. Thus the observed differences are more related to the different batches of mixtures than the CR contents. However, PAH pattern in the three leachates are very similar and are related to the asphalt pattern but not to the crumb rubber pattern.

**Fig. 7:**
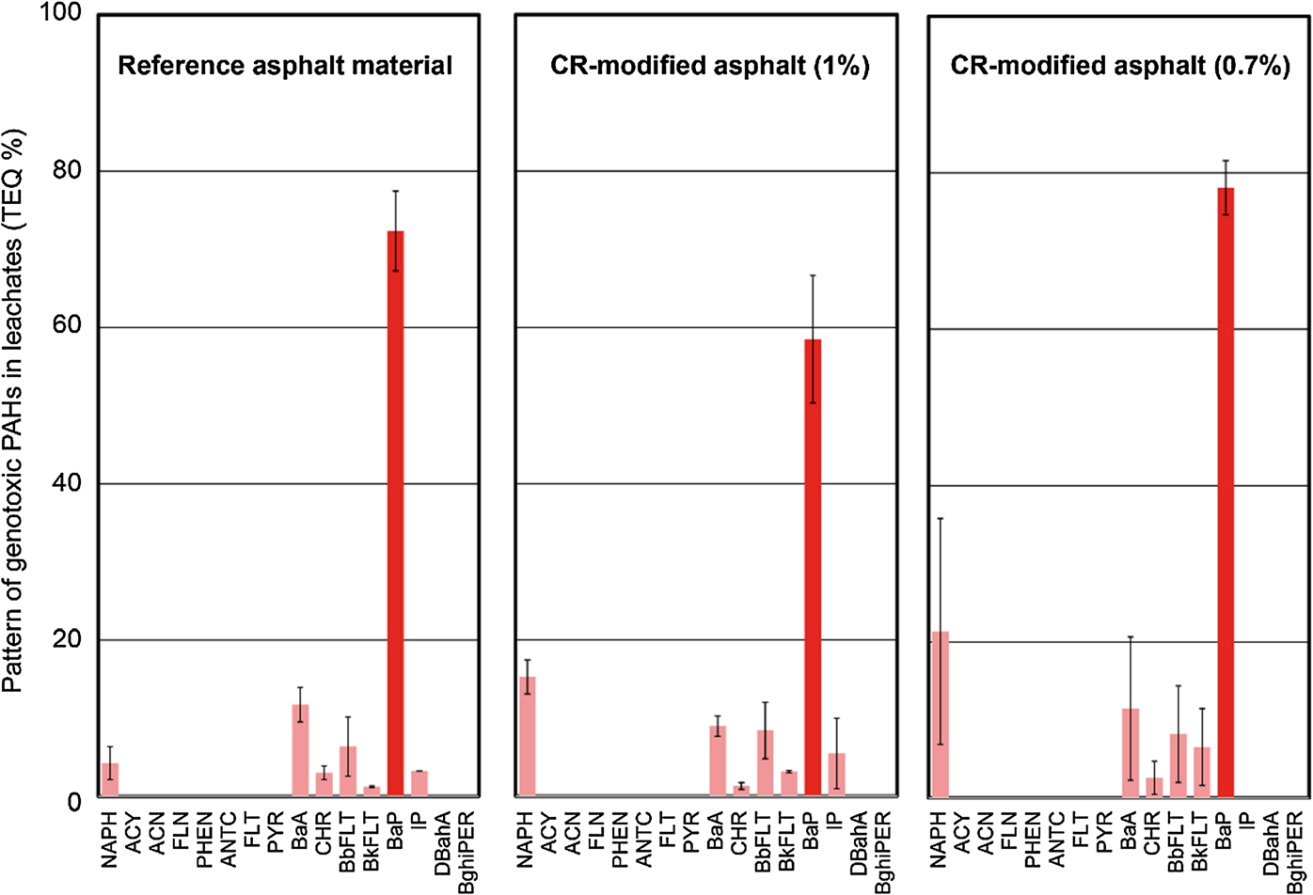
Proportions of eight genotoxic PAHs (TEQ-%) in asphalt leachates. Reference asphalt without CR and modified asphalts with 1% and 0.7% CR are compared. For abbreviations refer to Table 2

The genotoxic potential of these leachates can be compared from Fig. 7. Similar patterns are obtained, with benzo(a)pyrene (13) as the dominant genotoxic PAH along with contributions of naphtalene (1). There is no increase of the genotoxic potential due to the presence of CR either. The concentrations were found to be lower for the SDA mixtures with CR (1.0 and 0.9 mg TEQ/l) than for the reference mixture (4.3 mg TEQ/l).

**Fig. 8:**
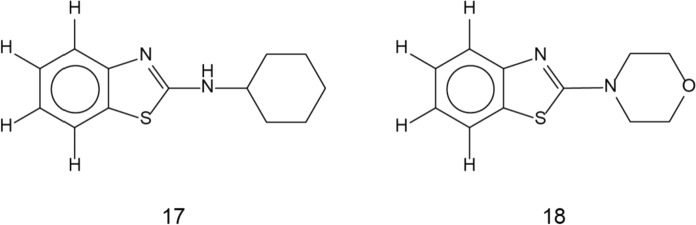
Chemical structures of two benzthiazole derivatives found in the CR material and leachates from CR-modified asphalts. N-cyclohexyl-amino-benzthiazole (17) and 2,4-morpholino-benzthiazole (18) were studied. For abbreviations refer to Table 2

In parallel, the CR and asphalt mixtures samples were checked for the presence of benthiazole (BT) compounds. Although various BT derivatives are used in the rubber industry, in this study cyclohexyl-amino-BT (17, HABT) and 2,4-morpholino-BT (18, MoBT) are used as rubber markers. Respective chemical formulas are given in Fig. 8.

These BT-derivatives have been identified before in CR and are well-known. Furthermore, isotope-labeled standard materials are availible to quantify them. Both rubber markers indeed could be identified in the type of CR used in this work. HABT- (17) and MoBT- (18) contents (ng/g) in organic CR extracts (DCM) and respective concentrations (ng/l) in aqueous leachates of different asphalt mixtures are shown in Table 3.

**Table 3:** Benzthiazole derivatives in organic CR-extracts (ng/g, *n* = 1) using dichloromethane and in leachates of non-modified and CR-modifed asphalts (ng/l, *n* = 3) using acidic water

	CR (ng/g)^a^	SDA-Ref (ng/l)^b^	SDA-CR 0.7% (ng/l)^b^	SDA-CR 1.0% (ng/l)^b^
MOBT (18)	810	10 ± 1	124 ± 4	169 ± 2
HABT (17)	690	2.1 ± 0.1	52 ± 3	61 ± 3

^a^Solid CR was extracted with DCM

^b^Leachate concentrations are reported based on the volumes of aqueous extracts

It is confirmed that MOBT (18) and HABT (17) are present in the CR material and are extractable with acidic water (pH = 4.93) from CR-modified asphalt mixtures with the given leaching procedure. Concentrations of MOBT (18) and HABT (17) in organic extracts of the CR sample were found to be similar (810 ng MOBT/g versus 690 ng HABT/g). As expected, substantially higher concentrations of these BT-compounds were found in aqueous leachates of CR-modified asphalt mixtures (Table 3). MOBT (18) concentrations of 10 ± 1, 124 ± 4 and 169 ± 2 ng/l were found in asphalt leachates without and with 0.7% and 1.0% CR, respectively. HABT (17) concentrations even increased by factors of 26 and 30 from 2.1 ± 0.1 to 53 ± 3 and 61 ± 3 ng/l from asphalt leachates without and with 0.7% and 1.0% CR. These values seem to be related to the amount of the CR in each asphalt mixture. Interesstingly, the MOBT/HABT ratios in leachates differ from the one observed for CR. In this case, the higher polarity of MOBT (18) results in an increased MOBT-concentration in the leachates with respect to HABT (17). It can be concluded that the leaching of MOBT (18) from the CR-modified asphalt is more efficient than the one of HABT (17). However, both BT-derivatives, which are more polar than PAHs, are released from CR-asphalt and can be detected in leachates and with it, will eventually be washed out from CR-modified asphalt roads. A washing step of the CR-material with acidic water, before its application in asphalt mixtures, could possibly lower the amounts of benzthiazoles in CR and in CR-modified asphalt and their release to the environment.

**Fig. 9:**
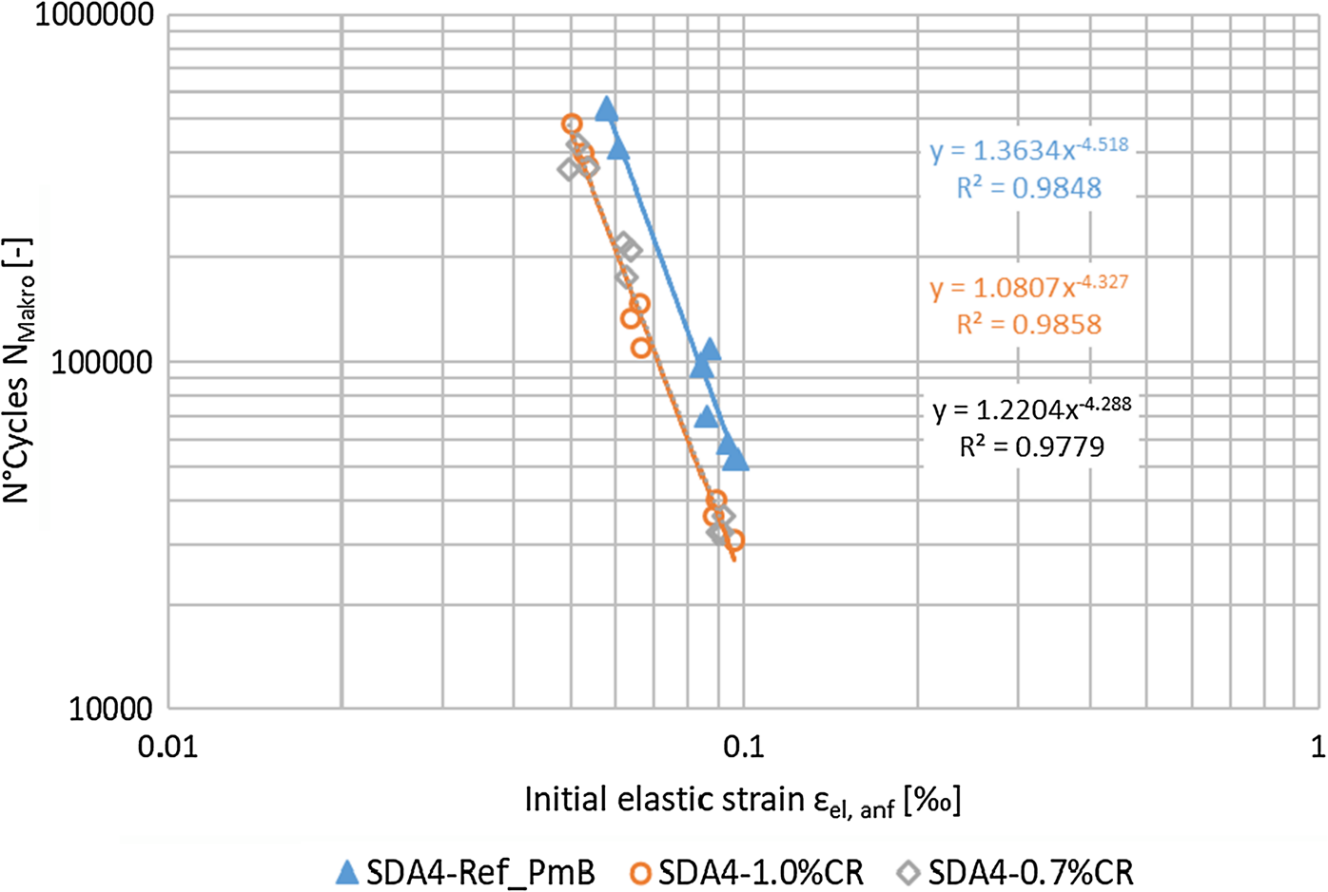
Fatigue testing results for three different loading amplitudes at *f* = 10 Hz and at a test temperature of 10 °C: loading cycles to fatigue failure *Nmacro* vs. initial strain level ε_el_. (ε_6, SDA4-Ref_PmB_ = 50.3 µm/m and ε_6, SDA4-%CR_ = 41.8 µm/m)

The mechanical performance of the two asphalt mixtures with CR was evaluated in comparison to the response obtained for the reference asphalt mixture prepared with PmB. Figure 9 shows the behaviour of the cylindrical specimens regarding their resistance to fatigue. It can be observed that the use of PmB (blue curve) offers a better response at high and low strain levels. Regarding the CR content (orange and gray curves), no significant difference was found between the two experimental mixtures. Furthermore, the classical parameter ε_6_, defined as the strain to reach one million cycles, was also calculated from the obtained fatigue data as ε_6, SDA4-Ref_PmB_ = 50.3 µm/m and ε_6, SDA4-%CR_ = 41.8 µm/m. Theses values confirmed that the resistance to the fatigue was worse for the CR mixtures. However, previous studies of SDA mixtures with similar voids content have shown a fatigue resistance for SDA mixtures of 33 µm/m [35] indicating a comparable fatigue performance of the mixtures in the current experiments.

**Fig. 10:**
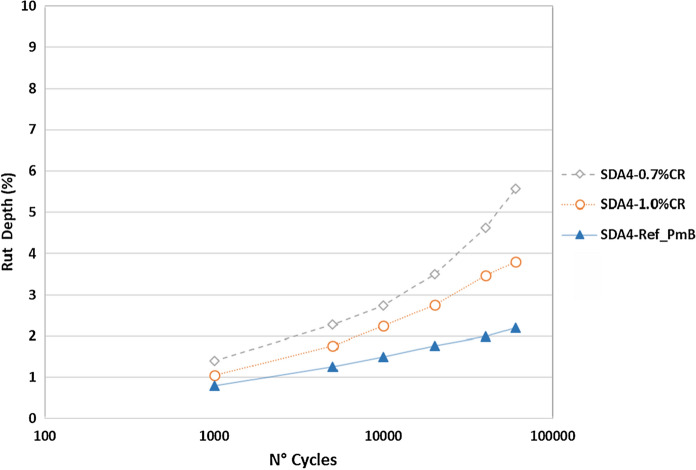
MMLS3 test results for the different asphalt mixtures at room temperature of ca 22 °C

The evolution of the rutting was monitored with the MMLS3 over 60,000 loading cycles. The values of rut depth for each slab compacted from the different mixtures are shown in Fig. 8. It was confirmed that conventional mixtures with PmB, designed for high performance, behave considerably better with very low rutting performance (< 2%). The CR mixtures also showed acceptable response against permanent deformation with maximum values after 60,000 cycles of 3.8% and 5.6% for the mixtures with 1.0% and 0.7% CR, respectively. The trends are similar to that previously reported for similar mixtures. In a previous study, it was shown that EC asphalt showed similar rutting performance to the ECR [36]. Although no requirements exist for this type of test on SDA mixtures, the conventional rutting tests requirements lie in the 7.5–10% range (SN 640-431-1c). Using these values as a guide indicates that the obtained rut depths for the CR modified mixtures are comparatively low. This fact anticipates that the incorporation of CR using the dry process would not compromise the performance of SDA mixtures against rutting at service temperatures (Fig. 10).

**Fig. 11:**
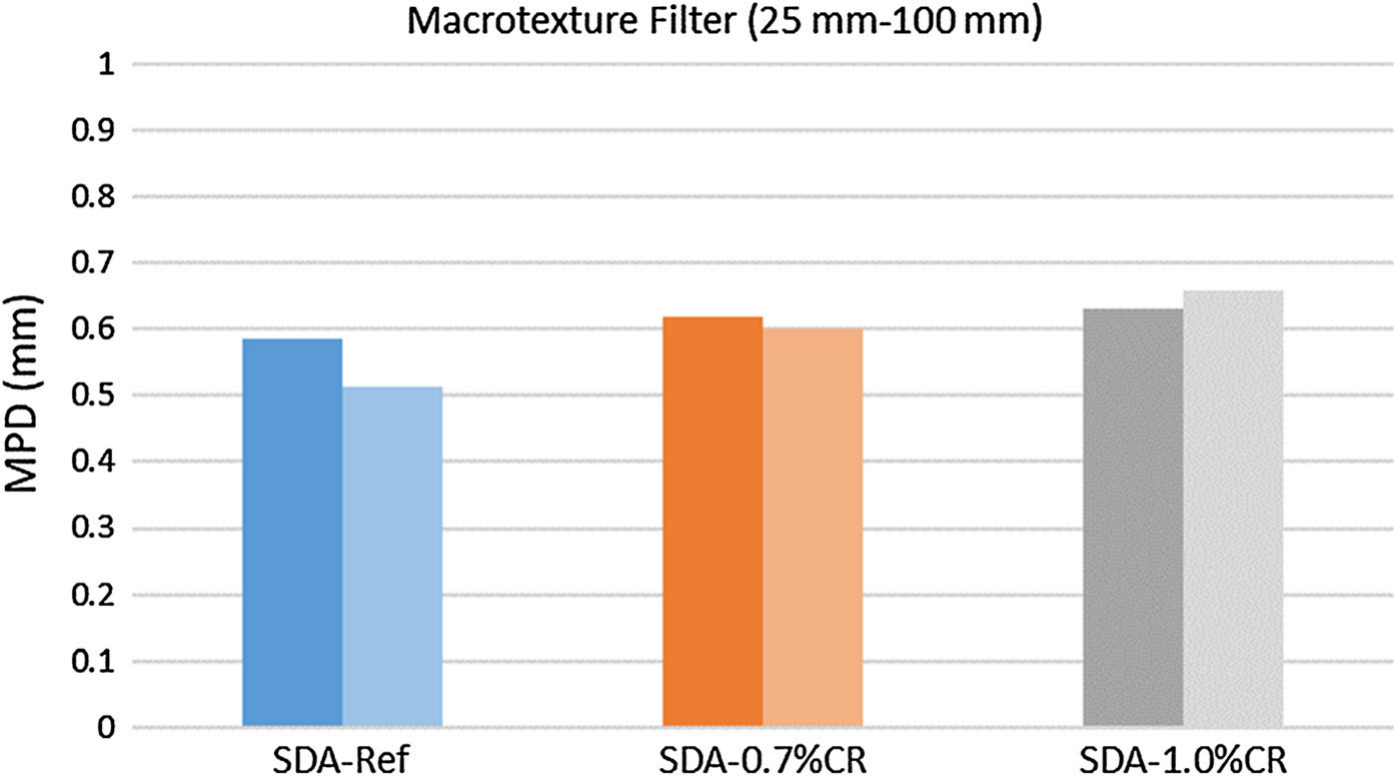
Surface texture levels before (dark color) and after wearing (light color) for the different asphalt mixtures

Finally, the properties of the surface texture of the different slabs were analyzed. The PTVs were measured to quantify skid resistance under wet conditions. In this case, higher values were obtained for the SDA mixtures with CR (PTV = 65 for SDA4-1.0%CR and PTV = 63 for SDA4-0.7%) in comparison to the reference one (PTV = 46). Some countries specify certain thresholds for the skid resistance. For example, a retained PTV of higher than 55 after the first two months of service is required in Italy [11]*.* The values obtained for CR modified mixtures are above these limits. However, it is important to remark that unlike field measurements, lab measurements were carried out on surfaces not subjected to any wearing process due to the tire friction. In addition, the MPD levels of the different surfaces were assessed (Fig. 11), showing a slight increase in MPD with CR, which was in contrast to previous studies on dense mixtures with dry process CR (Paje et al. 2010) [10]. The results show that after experiencing wearing from the MMLS3 test, the reference mixture with PmB and SDA with 0.7% CR showed an MPD decrease whereas the 1% CR showed a slight increase. Nevertheless, this change was not significant for both experimental mixtures with CR. These results confirm how the MPD may evolve differently with the modification of porous [37] or dense pavements [38–40].

**Fig. 12:**
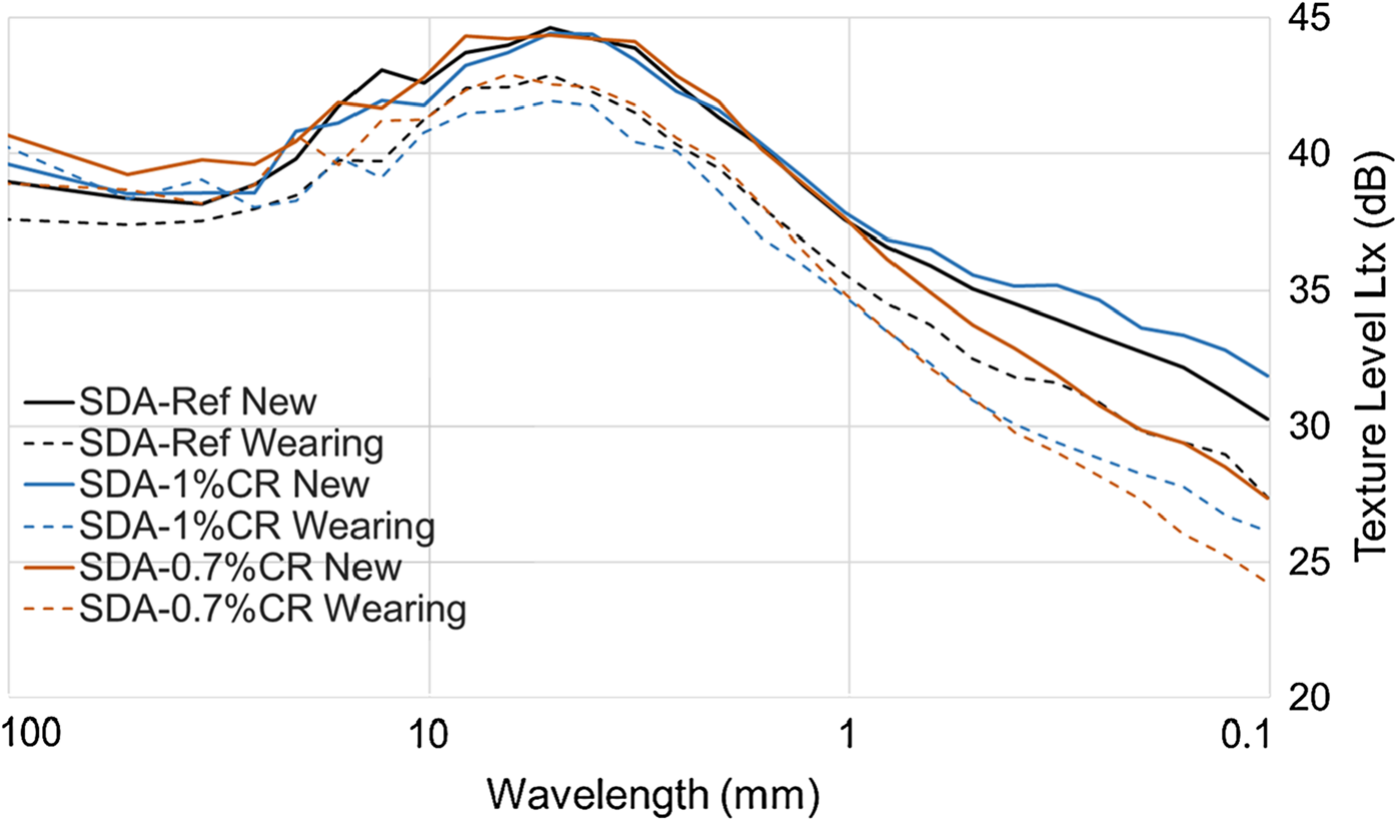
Texture level versus wavelength for the new surfaces and after exposure to loads under the wheel path (wearing)

The texture level profiles (Fig. 12) confirm that the samples are similar in macrotexture (> 1 mm), but the SDA-0.7%CR sample has significantly lower microtexture before wearing. After wearing, the texture level for all of the samples is reduced. The microtexture (< 1 mm) for the SDA-1.0%CR sample is especially lower after wearing, with a 5 dB reduction at 0.1 mm in wavelength, and a reduction in the texture level overall. In this case, the decrease in both the micro and macro texture was consistent with previous findings with dry process CR [10], showing texture level to me more reliable than MPD.

## Conclusions

Due to their high performance characteristics, conventional semi-dense asphalt (SDA) pavements required the use of polymer-modified binders in their mix design. However, the production and life cycle of these polymer-modified solutions can involve economic and environmental disadvantages. As an alternative, in this study, the potential use of crumb rubber (CR) from waste tires as additive for SDA mixtures using the dry process was assessed. Two contents of a CR type specially engineered for dry process applications were used to prepare different batches of SDA mixtures in an asphalt plant. Gaseous emissions during the production processes, leaching of CR additives with acidic water and performance tests as well as surface characterizations were carried out in order to evaluate the experimental SDA mixtures with CR and to compare them with a conventional SDA mixtures (with PmB). It was observed that the addition of CR had no negative effect on the overall emission of volatile organic compounds (VOC) and on the release of polycyclic aromatic hydrocarbons (PAH).

However, both investigated benzthiazole derivatives, which are derived from vulcanization additives used in the rubber production, were released from CR-modified asphalt under the given leaching conditions, which simulate exposure to acid rain. It is proposed that an additional washing step of the CR material, before mixing with asphalt, can remove these polar compounds and with it lower the risks for their release to the environment. Although the conventional mixture fabricated with PmB obtained better responses against fatigue and permanent deformation, the presence of CR in the mixtures did not compromise the mechanical behavior beyond requirements for this type of surfaces. Likewise, similar macrotexture levels were measured for all the slabs compacted from the different mixtures. Finally, the amount of CR incorporated and the binder content did not seem to have relevant influence on the factors evaluated in this study. Therefore, it can be concluded that the use of CR and its incorporation using the dry process could become a cheaper and environmentally friendly alternative even for high performance asphalt mixtures. The future construction of test tracks with these designs will be useful to further evaluate this technology since a more accurate VOC analysis during the production of larger amounts of mixtures with CR could be conducted and a field study of the final surface properties after compaction could be decisive to assess the importance of the differences found in micro-texture levels at lab scale.

## Data Availability

The raw/processed data required to reproduce these findings can be shared upon request.

## Abbreviations

BT: Benzothiazole
CR: Crumb rubber
DCM: Dichloromethane
GHG: Greenhouse gas
HABT: Cyclohexyl-amino-BT
MMLS3: Model mobile load simulator
MoBT: Morpholino-BT
MPD: Mean profile depth
PAH: Polycyclic aromatic hydrocarbon
PmB: Polymer modified bitumen
PSD: Power spectral density
PTV: Pendulum test value
RAP: Reclaimed asphalt pavement
SDA: Semi dense asphalt
SMA: Stone mastic asphalt
TEQ: Toxicity equivalence-weighted
TOC: Total organic compounds
VOC: Volatile organic compounds
